# Repeatability and Reproducibility of Axial and Lateral Measurements on Handheld Optical Coherence Tomography Systems Compared with Tabletop System

**DOI:** 10.1167/tvst.9.11.25

**Published:** 2020-10-21

**Authors:** Xi Chen, Vincent Tai, Brendan McGeehan, Gui-Shuang Ying, Christian Viehland, Ryan Imperio, Katrina P. Winter, William Raynor, Du Tran-Viet, Shwetha Mangalesh, Maureen G. Maguire, Cynthia A. Toth, for the BabySTEPS Group

**Affiliations:** 1 Department of Ophthalmology, Duke University, Durham, NC, USA; 2 Department of Ophthalmology, University of Pennsylvania, Philadelphia, PA, USA; 3 Department of Biomedical Engineering, Duke University, Durham, NC, USA

**Keywords:** optical coherence tomography, OCT, handheld OCT, central foveal thickness, RNFL thickness

## Abstract

**Purpose:**

To compare the repeatability and reproducibility of axial and lateral retinal measurements using handheld optical coherence tomography (OCT) systems and a tabletop OCT system.

**Methods:**

Graders measured central foveal thickness (CFT), optic nerve-to-fovea distance (OFD), and retinal nerve fiber layer (RNFL) thickness on OCT scans of the right eye of 10 healthy adults. Three OCT systems were used: handheld Leica Envisu, investigational handheld swept-source OCT (UC3), and Heidelberg Spectralis tabletop system. All eyes were imaged five times with each OCT system by each of two imagers. A components of variance analysis provided estimates of repeatability (variation due to random error) and reproducibility (variation due to imager, grader, and random error) expressed as standard deviation and (coefficient of variation %).

**Results:**

Repeatability of CFT (µm) for Envisu, UC3, and Spectralis was 5.9 (2.6%), 6.9 (2.9%), and 4.7 (2.1%), and the reproducibility was 6.1 (2.7%), 7.3 (3.1%), and 4.7 (2.1%), respectively. The repeatability of OFD (mm) was 0.13 (2.9%), 0.10 (2.3%), and 0.07 (1.6%), and the reproducibility was 0.13 (3.0%), 0.10 (2.3%), and 0.07 (1.6%,) respectively. The repeatability for RNFL thickness (µm) for Envisu, UC3, and Spectralis was 4.3 (7.8%), 2.7 (5.4%), and 2.9 (4.9%), and the reproducibility was 4.5 (8.3%), 2.9 (5.8%), and 2.9 (4.9%), respectively.

**Conclusions:**

All three OCT systems had good repeatability and reproducibility with coefficients of variation of less than 3.5% for CFT and OFD measurements, and less than 8.5% for RNFL thickness.

**Translational Relevance:**

Our findings inform the repeatability and reproducibility of retinal axial and lateral measurements on handheld OCT and are useful for both clinical research and patient care.

## Introduction

Since its inception in 1991, optical coherence tomography (OCT) has revolutionized the diagnosis and management of many eye diseases.^1^ Tabletop OCT systems have been shown to be repeatable and reproducible instruments, but these systems cannot be easily used on pediatric subjects.^2^^–^^8^ The development of handheld OCT and its adaptation for pediatric use has enabled imaging in a supine position, as well as in patients who could not comply with positioning for tabletop imaging.^9^ However, there have been limited studies examining the repeatability and reproducibility of handheld OCT systems.^10^

Three quantitative values on OCT are often measured in pediatric eye research and clinical studies: central foveal thickness (CFT), optic nerve-to-fovea distance (OFD), and retinal nerve fiber layer (RNFL) thickness. CFT is an axial measurement, OFD is a lateral measurement, and RNFL thickness depends on both to obtain thickness at specific lateral locations from the optic nerve head. Factors that could influence real-world CFT measurements include image quality, image tilt, foveal frame selection, variable retinal layer reflectance from different OCT systems, and segmentation error. Lateral measurements on OCT are further affected by subject eye length, scan size, image rotation (variable dependence on fast and slow axis), imager hand motion, and subject body position (sitting or supine).^9^ RNFL thickness measurements can have variability caused by the factors affecting either axial and lateral OCT measurements.^11^

Our group has previously adapted a commercial handheld OCT system for infant use (Envisu C2300; Leica Microsystems, Buffalo Grove, IL) and has subsequently developed our current research handheld OCT system (UC3). These handheld OCT systems have been used in our ongoing infant retinal imaging study (BabySTEPS, NCT02887157). We are unable to conduct rigorous reproducibility studies with multiple repetitions and multiple research systems in the pediatric vulnerable population. Our goal of the current study was to evaluate the repeatability and reproducibility of these handheld OCT systems compared with a tabletop OCT system (Spectralis, Heidelberg Engineering, Heidelberg, Germany) in healthy adult volunteers. In addition, we evaluated the variance induced by body position (sitting vs. supine) of handheld OCT measurements in adult volunteers.

## Methods

### Study Protocol

This study was approved by the Duke University Health System institutional review board and adhered to the tenets of the Declaration of Helsinki. Informed consent was obtained from the subjects after explanation of the nature, risks, and benefits of the study. We conducted a prospective imaging study in 10 healthy adult volunteers with minimal refractive error (spherical equivalent between −2 and +2 diopters) to compare the repeatability and reproducibility of OCT measurements of the two handheld systems (Envisu [Leica Microsystems], 32 kHz spectral domain OCT, 860 nm, 10 × 10 mm, 1000 A-scans/B-scan, 100 B-scans/volume; and investigational handheld swept-source UC3 system, 200 kHz swept source OCT, 1060 nm, 10.46 × 10.46 mm, 951 A-scans/B-scan, 256 B-scans averaged at 128 locations/volume) to the tabletop system (Spectralis [Heidelberg Engineering], 40 kHz spectral domain OCT, 870 nm, 30° scan, 768 A-scans/B-scan, 61 B-scans/volume, averaged 7–11 times per B-scan location). For each volunteer, two imagers (DT and XC) obtained five sets of volume scans in the right eye with each of the three imaging systems. Each set of volume scans had two volumes per set in the Envisu, five volumes per set in UC3, and one volume per set in Spectralis. For each system, 10 volume scans (five volumes for each imager) containing both the optic disc and fovea were selected for CFT, OFD, and RNFL thickness measurements (Supplementary Fig. 1). Envisu and Spectralis imaging was conducted on the first day. UC3 imaging was conducted on the second day at a similar time of the day (within +/− 3 hours).

To evaluate the variance induced by positional changes (sitting vs. supine), one imager (DT) obtained five sets of volume scans in the left eye of three healthy adult volunteers in sitting position and five sets of volume scans in supine position with Envisu and UC3. Scans for CFT and OFD measurements were selected as noted earlier.

### Image Analysis

Automatic segmentation of each selected volume was performed using custom MATLAB (MathWorks, Inc., Natick, MA) code (DOCTRAP).^12^ The foveal scan was selected and marked by each of the two graders who also manually corrected the segmentation as needed independently (KPW and XC). CFT was determined by the distance between internal limiting membrane and Bruch's membrane (Supplementary Fig. 2). OFD was determined from the markings of the fovea and optic disc by three graders independently (KPW, XC, and WR) and calculated using custom MATLAB code. The RNFL was further segmented using DOCTRAP with manual correction by one grader (KPW). The RNFL thickness at 1.7 mm from the optic disc (the average thickness of the papillomacular bundle, the temporal RNFL at 1.7 mm from the optic nerve along the 30° arc centered on the axis from optic nerve to fovea) was determined using custom MATLAB code as previously described.^11^

### Statistical Analysis

Following the principles of reliability analysis,^13^ we analyzed data using analysis of variance to estimate components of variation due to OCT system, grader, imager, subject, position, and random error. Repeatability refers to the variation due to random error, whereas reproducibility refers to the variation due to random error, imager, grader, and/or position. We calculated the estimate of repeatability standard deviation (SD) as the square root of the repeatability variance. We calculated the reproducibility SD as the square root of the reproducibility variance and the coefficient of variation (%CV) as the percent of SD divided by the mean of the measure. The intraclass correlation (ICC) was reported as the proportion of between-subject variance compared with the total variance. All statistical analyses were performed in SAS v9.4 (SAS Institute Inc, Cary, NC).

## Results

The 10 participants had an average age (SD) of 37.9 (10.4) years with minimal refractive error (spherical equivalent between −2 and +2 diopters). Eight participants were women. All were healthy adult volunteers with no known eye issues or history of ocular surgeries.

### Repeatability and Reproducibility of CFT

Out of the 300 scans (100 scans with 3 OCT systems) that were graded independently by 2 graders, 13 scans (2.2%, 9 Envisu, 1 UC3, and 3 Spectralis; 2 grader 1 and 11 grader 2) were ungradable due to image quality or an image being cropped and 587 scans (97.8%) were analyzed. The mean (SD) of CFT across all three OCT systems was 227.3 (16.1) µm. For Envisu, UC3, and Spectralis, the mean (SD) of CFT was 223.0 (14.7), 236.0 (15.8), and 223.0 (13.9) µm, respectively. For CFT overall, repeatability was 6.2 µm (2.72%) and reproducibility was 9.7 µm (4.28%) (Table 1). The repeatability of CFT for Envisu, UC3, and Spectralis was 5.9 µm (2.63%), 6.9 (2.90%), and 4.7 (2.11%) µm, respectively. The reproducibility for Envisu, UC3, and Spectralis was 6.1 (2.72%), 7.3 (3.09%), and 4.7 (2.13%) µm, respectively (Table 2).

**Table 1.** Results of the Components of Variance Analysis for Estimating the Overall Repeatability and Reproducibility of CFT and OFD Measurements Across all Three OCT Systems

	CFT (µm)^a^				OFD (mm)^a^			
Source of Variation	Variance	SD	Coefficient of Variation (%)	ICC (%)	Variance	SD	Coefficient of Variation (%)	ICC (%)
Subject	200.3	14.2	6.23	67.94	0.13	0.37	8.39	86.67
Imager	0.2	0.5	0.20	0.07	0.00	0.01	0.13	0.00
Grader	0.3	0.5	0.22	0.10	0.00	0.02	0.39	0.00
Device system	55.9	7.5	3.29	18.96	0.00	0.04	1.01	0.00
**Repeatability**								
Residual error	38.1	6.2	2.72	12.92	0.02	0.13	2.90	13.33
**Reproducibility**								
Sum of imager, grader, device system and residual error	94.5	9.7	4.28	32.06	0.02	0.14	3.10	13.33

a There were 587 CFT gradings (13 ungradable) and 866 OFD gradings (34 ungradable).

**Table 2.** Results of the Components of Variance Analysis for Estimating the Repeatability and Reproducibility of CFT and OFD Measurements for Envisu, UC3, and Spectralis OCT Systems

		CFT (µm)^a^				OFD (mm)^a^			
OCT System	Source of Variation	Variance	SD	Coefficient of Variation (%)	ICC (%)	Variance	SD	Coefficient of Variation (%)	ICC (%)
**Envisu**	Subject	199.0	14.1	6.33	84.43	0.16	0.40	9.04	88.89
	Imager	2.4	1.6	0.69	1.02	0.00	0.03	0.60	0.00
	Grader	0.0	0.0	0.00	0.00	0.00	0.01	0.32	0.00
	**Repeatability**								
	Residual error	34.3	5.9	2.63	14.55	0.02	0.13	2.92	11.11
	**Reproducibility**								
	Sum of imager, grader, and residual error	36.7	6.1	2.72	15.57	0.02	0.13	3.00	11.11
**UC3**	Subject	222.0	14.9	6.31	80.58	0.15	0.38	8.80	93.75
	Imager	0.1	0.3	0.16	0.04	0.00	0.00	0.00	0.00
	Grader	6.4	2.5	1.07	2.32	0.00	0.02	0.53	0.00
	**Repeatability**								
	Residual error	47.0	6.9	2.90	17.06	0.01	0.10	2.27	6.25
	**Reproducibility**								
	Sum of imager, grader, and residual error	53.5	7.3	3.09	19.42	0.01	0.10	2.33	6.25
**Spectralis**	Subject	186.0	13.6	6.16	89.21	0.12	0.35	7.90	92.31
	Imager	0.3	0.5	0.25	0.14	0.00	0.00	0.00	0.00
	Grader	0.0	0.0	0.00	0.00	0.00	0.01	0.19	0.00
	**Repeatability**								
	Residual error	22.2	4.7	2.11	10.65	0.01	0.07	1.63	7.69
	**Reproducibility**								
	Sum of imager, grader, and residual error	22.5	4.7	2.13	10.79	0.01	0.07	1.64	7.69

a There were 587 CFT gradings (13 ungradable) and 866 OFD gradings (34 ungradable).

Imager variation was the smallest for UC3 (0.3 µm, 0.16%) compared with Envisu (1.6 µm, 0.69%) and Spectralis (0.5 µm, 0.25%). Grader variation only occurred in UC3 (2.5 µm, 1.07%) for gradable scans (Table 2). The calculated ICC for CFT was 0.84 (Envisu), 0.81 (UC3), and 0.89 (Spectralis), respectively.

### Repeatability and Reproducibility of Optic Disc-to-Fovea Distance

Out of the 300 scans (100 scans with 3 OCT systems) that were graded independently by 3 graders, 34 scans (3.8%, 25 Envisu, 4 UC3, and 5 Spectralis; 7 grader 1, 11 grader 2, and 16 grader 3) were ungradable due to optic disc and fovea not well-identified or not both included and 866 scans (96.2%) were analyzed. The mean (SD) of OFD across all three OCT systems was 4.37 (0.37) mm. For Envisu, UC3, and Spectralis, the mean (SD) was 4.41 (0.40), 4.33 (0.38), and 4.38 (0.34) mm, respectively. For OFD overall, repeatability was 0.13 mm (2.9%) and reproducibility was 0.14 mm (3.1%) (Table 1). The repeatability of OFD for Envisu, UC3, and Spectralis was 0.13 (2.92%), 0.10 (2.27%), and 0.07 (1.63%) mm, respectively. The reproducibility for Envisu, UC3, and Spectralis was 0.13 (3.00%), 0.10 (2.33%), and 0.07 (1.64%) mm, respectively (Table 2).

Imager variation was the smallest for Spectralis (0.00 mm, 0.00%) and UC3 (0.00 mm, 0.00%), and larger for Envisu (0.03 mm, 0.60%). Grader variation was the smallest for Envisu (0.01 mm, 0.32%) and Spectralis (0.01 mm, 0.19%), and slightly larger for UC3 (0.02 mm, 0.53%) for gradable scans (Table 2). The calculated ICC for OFD was 0.90 (Envisu), 0.93 (UC3), and 0.96 (Spectralis), respectively.

### Effect of Body Position on CFT and OFD Measurements

To evaluate the variance induced by positional change, handheld OCT imaging was performed in the left eye of three adult volunteers using Envisu and UC3. For CFT, out of the 60 scans graded by 2 graders, 9 scans (7.5%, 3 supine and 6 sitting) were ungradable due to image quality and unable to determine CFT and 111 scans (92.5%) were analyzed. The Envisu mean (SD) was 234.0 (24.0) µm at supine position and 229.0 (15.5) µm at sitting position, and the UC3 mean (SD) was 247.0 (20.5) µm at supine position and 245.0 (20.3) µm at sitting position. The position variation (CV) was larger for Envisu (3.1 µm, 1.36%) than UC3 (0.0 µm, 0.00%) (Table 3).

**Table 3.** Results of the Components of Variance Analysis and the Repeatability and Reproducibility of CFT and OFD Measurements for Envisu, UC3, and Spectralis OCT Systems with Positional Change

		CFT (µm)^a^				OFD (mm)^a^			
OCT System	Source of Variation	Variance	SD	Coefficient of Variation (%)	ICC (%)	Variance	SD	Coefficient of Variation (%)	ICC (%)
**Envisu**	Subject	517.6	22.8	9.83	89.75	0.03	0.18	4.17	50.00
	Grader	0.0	0.0	0.00	0.00	0.01	0.09	2.18	16.67
	Position	9.9	3.1	1.36	1.72	0.00	0.03	0.68	0.00
	**Repeatability**								
	Residual error	49.2	7.0	3.03	8.53	0.02	0.13	3.08	33.33
	**Reproducibility**								
	Sum of grader, position, and residual error	59.1	7.7	3.32	10.25	0.03	0.16	3.83	50.00
**UC3**	Subject	537.1	23.2	9.41	90.13	0.03	0.17	3.94	50.00
	Grader	23.5	4.8	1.97	3.94	0.01	0.08	1.99	16.67
	Position	0.0	0.0	0.00	0.00	0.00	0.00	0.00	0.00
	**Repeatability**								
	Residual error	35.3	5.9	2.41	5.92	0.02	0.14	3.18	33.33
	**Reproducibility**								
	Sum of grader, position, and residual error	58.8	7.7	3.11	9.87	0.02	0.16	3.75	33.33

a There were 111 CFT gradings (9 ungradable) and 166 OFD gradings (14 ungradable).

For OFD, out of 60 scans graded by 3 graders, 14 scans (7.8%, 5 supine and 9 sitting) were ungradable due to image quality or unable to determine both fovea and optic nerve and 166 scans (92.2%) were analyzed. The Envisu mean (SD) was 4.30 (0.20) mm at supine position and 4.36 (0.23) mm at sitting position, and the UC3 mean (SD) was 4.28 (0.20) mm at supine position and 4.25 (0.21) mm at sitting position. UC3 again yielded smaller position variation (CV) of 0.00 mm (0.00%) compared with 0.03 mm (0.68%) for Envisu (Table 3). We attempted to compare the CFT and OFD measurements at supine versus sitting position. However, we observed inconsistent results for each of the three participants. The sample size (three subjects, five attempted scan sets at sitting or supine position) was too small to draw a definitive conclusion if there is a significant difference in OCT measurements with positional change.

### Repeatability and Reproducibility of RNFL Thickness

Out of the 300 scans graded by 1 grader, 16 scans (5.3%, 13 Envisu, 2 UC3, and 1 Spectralis) were ungradable due to optic disc and fovea not well-identified or not both included, poor image quality, image crop, or incomplete coverage of the area of RNFL measurement and 284 scans (94.7%) were analyzed. The mean (SD) of RNFL thickness across all three OCT systems was 54.8 (11.2) µm. For Envisu, UC3, and Spectralis, the mean (SD) was 54.3 (12.2), 50.3 (9.9), and 59.6 (9.5) µm, respectively. For RNFL thickness overall, repeatability was 3.8 µm (7.00%) and reproducibility was 6.2 µm (11.40%) (Table 4). The repeatability for Envisu, UC3, and Spectralis was 4.3 µm (7.84%), 2.7 µm (5.42%), and 2.9 µm (4.86%), respectively (Table 5). The reproducibility for Envisu, UC3, and Spectralis was 4.5 µm (8.25%), 2.9 µm (5.83%), and 2.9 µm (4.86%), respectively. Imager variation was the smallest for Spectralis (0.0 µm, 0.00%), and larger for Envisu (1.4 µm, 2.56%) and UC3 (1.1 µm, 2.15%). The calculated ICC for RNFL thickness was 0.88 (Envisu), 0.92 (UC3), and 0.91 (Spectralis), respectively (Table 5).

**Table 4.** Results of the Components of Variance Analysis and the Overall Repeatability and Reproducibility of RNFL Thickness Measurements Across all Three OCT Systems

	RNFL Thickness (µm)^a^			
Source of Variation	Variance	SD	Coefficient of Variation (%)	ICC (%)
Subject	104.7	10.2	18.68	72.86
Imager	0.8	0.9	1.64	0.56
Device system	23.5	4.8	8.84	16.35
**Repeatability**				
Residual error	14.7	3.8	7.00	10.23
**Reproducibility**				
Sum of Imager, device system and residual error	39.0	6.2	11.40	27.14

a There were 284 RNFL thickness gradings (16 ungradable).

**Table 5.** Results of the Components of Variance Analysis and the Repeatability and Reproducibility of RNFL Thickness Measurements for Envisu, UC3, and Spectralis OCT Systems

		RNFL Thickness (µm)^a^			
OCT System	Source of Variation	Variance	SD	Coefficient of Variation (%)	ICC (%)
**Envisu**	Subject	142.5	11.9	21.97	87.64
	Imager	1.9	1.4	2.56	1.17
	**Repeatability**				
	Residual error	18.2	4.3	7.84	11.19
	**Reproducibility**				
	Sum of imager, grader, and residual error	20.1	4.5	8.25	12.36
**UC3**	Subject	98.1	9.9	19.71	91.94
	Imager	1.2	1.1	2.15	1.12
	**Repeatability**				
	Residual error	7.4	2.7	5.42	6.94
	**Reproducibility**				
	Sum of imager, grader, and residual error	8.6	2.9	5.83	8.06
**Spectralis**	Subject	89.3	9.4	15.84	91.40
	Imager	0.0	0.0	0.00	0.00
	**Repeatability**				
	Residual	8.4	2.9	4.86	8.60
	**Reproducibility**				
	Sum of imager, grader, and residual error	8.4	2.9	4.86	8.60

a There were 284 RNFL thickness gradings (16 ungradable).

## Discussion

Our current study examined the repeatability and reproducibility of axial and lateral retinal measurements of two handheld OCT systems (Envisu and UC3) compared with a tabletop OCT system (Spectralis). We found that for both CFT and OFD measurements, all systems yielded a repeatability and reproducibility coefficient of variation of less than 3.5%. When compared with the handheld systems, the tabletop Spectralis system did yield a smaller variance for both repeatability and reproducibility of each measure. For all systems, RNFL thickness exhibited more variability than either CFT or OFD, as evident by the higher value of residual coefficient of variation, as it was dependent on variations from both axial and lateral measurements. For RNFL thickness, Spectralis again yielded a smaller variance, whereas all systems yielded a coefficient of variation of less than 8.5% for both repeatability and reproducibility. Both CFT and RNFL thickness are primary outcomes for the ongoing BabySTEPS study. For all three values (CFT, OFD, and RNFL thickness), all three OCT systems had strong repeatability as represented by ICC values above 0.80, which reflected the small variation due to other components and an ability to detect changes between subjects.

Compared with tabletop systems, handheld OCT has more potential sources of variance. First, handheld systems are often used on infants or children who do not fixate. It is frequent to observe artifacts due to eye movement, saccades, and hand movement. Second, it can be difficult to maintain a particular posture through the duration of a volume acquisition, which may be ameliorated by increasing the speed of acquisition. Third, there is a higher likelihood of image rotation as it is more difficult to determine the eye alignment. Despite this, a recent retrospective study from our group noted similar reproducibility in handheld OCT in infants compared with tabletop OCT in adult volunteers.^10^ Our current prospective study showed that handheld OCT systems produced repeatable and reproducible measurements and there was little variation contributed by different imagers or graders. However, the tabletop Spectralis system, as expected, did outperform the handheld Envisu and UC3 systems for all three measurements. Thus tabletop OCT instruments may still be preferred over handheld instruments in clinical studies or trials when possible.

Axial measurement is a property of the laser and is less affected by hand/patient eye movement during the acquisition process. However, it is plausible that the wavelength difference (Envisu and Spectralis use spectral-domain OCT engines [860, 870 nm] and UC3 uses a swept-source OCT engine [1060 nm] may lead to different optical scattering of retinal layers, and thus lead to different perceived layer thicknesses. Similarly, although the segmentation software DOCTRAP was fully objective, it may exhibit variable effects on the position of the segmentation line of different retinal layers. Our data found similar CFT values between Envisu and Spectralis, whereas CFT value measured by UC3 was approximately 5% larger. This indicates that a conversion factor for CFT measurements may be considered when comparing measurements between Envisu and UC3 in healthy adult volunteers. However, the conversion factor may be specific to its subject population. Thus a different conversion factor may be needed prior to application to infants or patients with diabetic macular edema. Additionally, swept-source OCT has a lower axial resolution compared with spectral domain OCTs. This may also contribute to the slightly lower repeatability and reproducibility (approximately 0.3%–1.0% difference) of CFT measured by UC3 compared with Envisu and Spectralis.

Lateral measurements, however, depend on the axial length of the eye and the reference arm length, as well as the inherent eye model length of the respective OCT device.^9^^,^^14^ Image rotation could also affect lateral measurements. When obtaining an OCT volume, the fast axis (along the B-scan) is less affected by hand and/or patient eye movements compared with the slow axis (perpendicular to the B-scan direction). Thus a higher degree of image rotation away from the fovea to optic nerve axis will introduce more variance due to a larger slow axis component. UC3, using a 200 kHz engine, is approximately 6 times faster than Envisu (32 kHz engine). This would explain why UC3 has a better repeatability and reproducibility, as well as a lower percentage of ungradable scans, for both OFD and RNFL thickness measurements than Envisu.

RNFL thicknesses have added complexity as they rely on both axial and lateral OCT measurements, especially in infant and pediatric eyes that are changing in size. To longitudinally follow the RNFL thickness, one may consider a different approach of measuring RNFL thicknesses: rather than a fixed distance from the optic disc, a proportion of distance between optic disc and the fovea may be considered. This may ameliorate the need to model the change in axial length and its related change in scan size.

The results from our study needs to be interpreted with limited generalizability, as it was conducted in healthy, compliant adult volunteers with minimal refractive error, and not the typical patient population in which handheld OCTs are commonly used (children, bed-ridden patients, and patients unable to cooperate with tabletop imaging). Infants and children, with limited ability of fixation and varying axial length through different stages of development, will have inherently more variability in OCT measurements. Limitations of our study include the small sample size that failed to compare the difference of OCT measurements with positional changes and the number of observers selected for each retinal measurement. As the goal of the study was to evaluate the handheld OCT devices compared with tabletop device and their utilization in BabySTEPS, the limited number of observers may limit its inference to retinal measurements of these devices in the general population by any observer.^15^

## Conclusions

Our study informs us that handheld OCT systems are repeatable and reproducible instruments to measure CFT, OFD, and RNFL thicknesses in healthy adult volunteers. This result will be applicable to current and future research studies and clinical practice using handheld OCT systems to evaluate changes in retinal measurements.

## Supplementary Material

Supplement 1
