# Supplementary material for: Repeatability and Reproducibility of Axial and Lateral Measurements on Handheld Optical Coherence Tomography Systems Compared with Tabletop System
Source: Transl Vis Sci Technol. 2020 Oct 21;9(11):25. doi: 10.1167/tvst.9.11.25 (PMC7585396; doi:10.1167/tvst.9.11.25)
Supplement: Supplement 1 [file tvst-9-11-25_s001.pdf]

Supplementary Figures

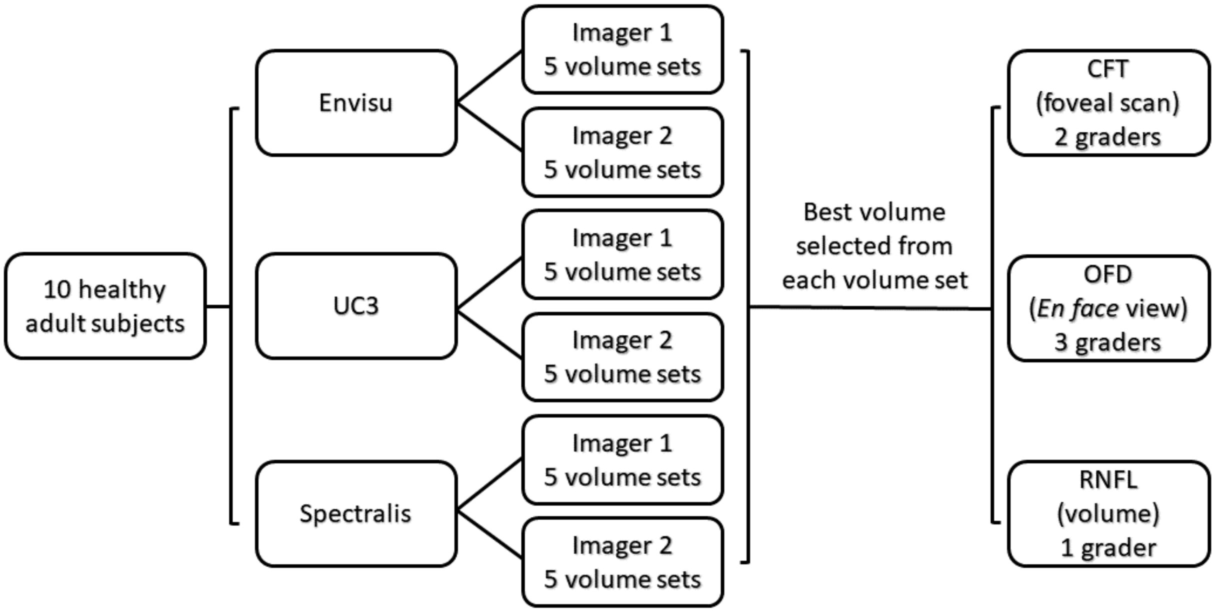

Supplementary Figure 1. Repeatability and reproducibility study flow diagram

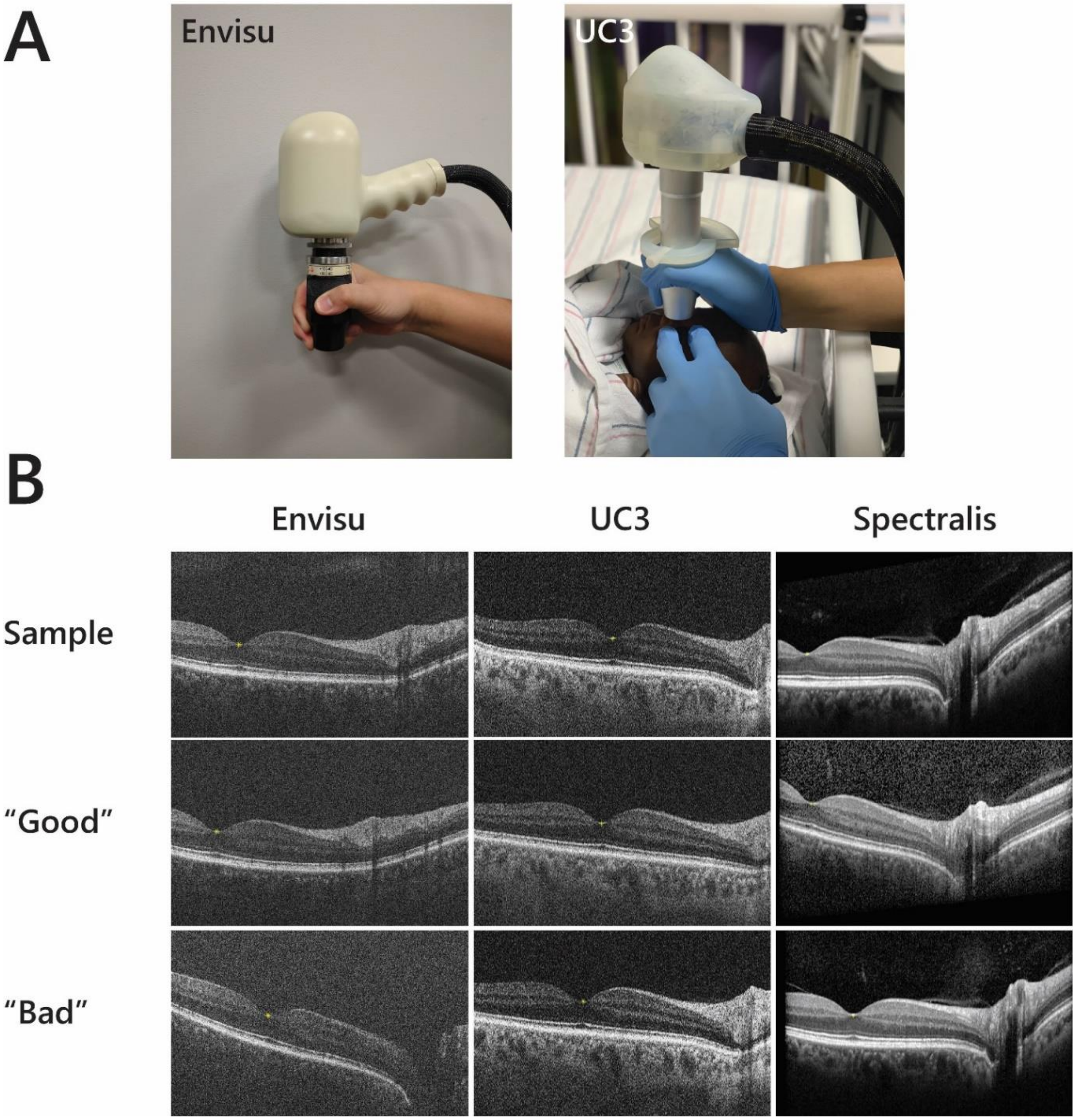

Supplementary Figure 2. Handheld OCT devices (Envisu spectral domain OCT and investigational UC3 swept source OCT) (A) used in the study and samples of OCT images from Envisu, UC3 and Spectralis from the same eye, with good or bad agreement of central foveal thickness values relative to the sample images (B). Foveal frames were selected by the graders and locations of the fovea were marked with yellow asterisks. The sample image had a central foveal thickness value that was close to the mean for this eye. Images shown with "good" agreement had a central foveal thickness value that was closest to the value for the sample image, and images with "bad" agreement had a value furthest from that of the selected image. Note that the images were cropped to scale. Note that Envisu B-scans were not averaged; the UC3 B-scans were averaged 2x; and the Spectralis B-scans were averaged 7-11x.

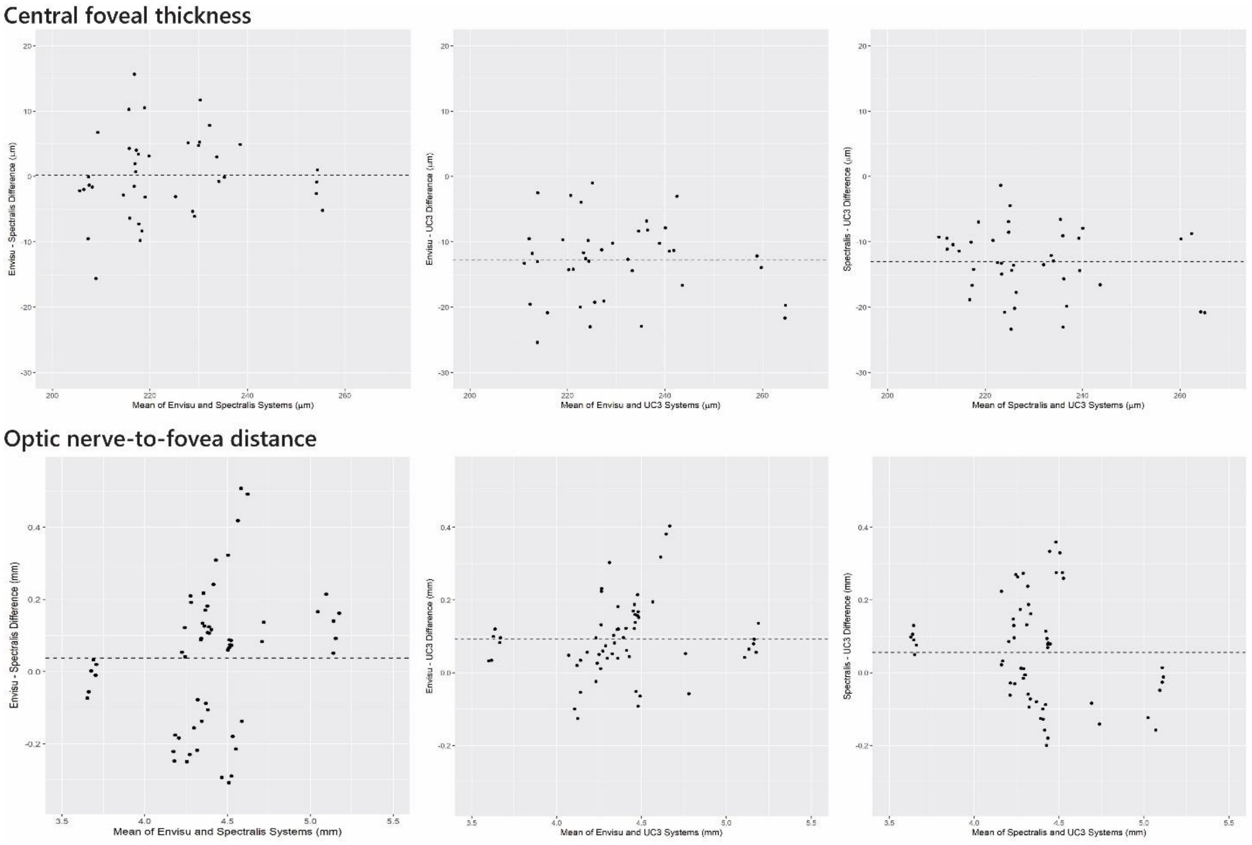

Supplementary Figure 3. Bland-Altman plots for inter-instrument analysis of central foveal thickness and optic nerve-to-fovea distance.
